# Knowledge and Theme Discovery across Very Large Biological Data Sets Using Distributed Queries: A Prototype Combining Unstructured and Structured Data

**DOI:** 10.1371/journal.pone.0080503

**Published:** 2013-12-02

**Authors:** Uma S. Mudunuri, Mohamad Khouja, Stephen Repetski, Girish Venkataraman, Anney Che, Brian T. Luke, F. Pascal Girard, Robert M. Stephens

**Affiliations:** 1 Advanced Biomedical Computing Center, Information Systems Program, SAIC-Frederick, Inc., Frederick National Laboratory for Cancer Research, Frederick, Maryland, United States of America; 2 Oracle Corporation, Reston, Virginia, United States of America; Leuven University, Belgium

## Abstract

As the discipline of biomedical science continues to apply new technologies capable of producing unprecedented volumes of noisy and complex biological data, it has become evident that available methods for deriving meaningful information from such data are simply not keeping pace. In order to achieve useful results, researchers require methods that consolidate, store and query combinations of structured and unstructured data sets efficiently and effectively. As we move towards personalized medicine, the need to combine unstructured data, such as medical literature, with large amounts of highly structured and high-throughput data such as human variation or expression data from very large cohorts, is especially urgent. For our study, we investigated a likely biomedical query using the Hadoop framework. We ran queries using native MapReduce tools we developed as well as other open source and proprietary tools. Our results suggest that the available technologies within the Big Data domain can reduce the time and effort needed to utilize and apply distributed queries over large datasets in practical clinical applications in the life sciences domain. The methodologies and technologies discussed in this paper set the stage for a more detailed evaluation that investigates how various data structures and data models are best mapped to the proper computational framework.

## Introduction

Ever since the original protein and nucleic acid database versions were supplied in book form and later distributed as a series of floppy disks, the biological sciences field has recognized a need for databases to store information. For many years, different types of biological data have been represented in standard relational databases, which form the basis of numerous searchable online databases spanning multiple biomedical domains [1], [2].

Most of these databases are available for download as tab delimited files. To accommodate these diverse data sources within the defined schemas required for a relational framework, various data normalization approaches that force the data to fit into the designated structures have been utilized. In order to maintain relations and allow knowledge mining, some of the popular biological databases have also become available in XML format (eXtensible Markup Language) (http://www.uniprot.org/docs/uniprot.xsd, http://www.nlm.nih.gov/bsd/licensee/elements_descriptions.html) and other tag-based hierarchical formats like ASN.1 (Abstract Syntax Notation One) (http://www.ncbi.nlm.nih.gov/Sitemap/Summary/asn1.html). More recently, large databases like UniProt have made their databases available for download in the RDF (Resource Description Framework) format (ftp://ftp.uniprot.org/pub/databases/uniprot/current_release/rdf/), which is more suitable for knowledge representation.

The accessibility and usability of these powerful resources has been further increased through the adoption of programmatic APIs, web services and direct access language packages (http://www.ncbi.nlm.nih.gov/entrez/query/static/esoap_help.html, http://www.rcsb.org/pdb/software/soap.do, http://useast.ensembl.org/info/docs/api/index.html, http://www.biomart.org). Consequently, it is now possible to dynamically combine the results from varied queries in different databases stored in an in-house data warehouse [3] or across the internet [4], [5] into a single result report in an automated manner.

In addition to these biological annotation databases, vast amounts of information is currently available through the very large and complex data sets produced by many research projects, including TCGA (http://cancergenome.nih.gov/), ICGC (http://icgc.org/), and 1000 genomes (http://www.1000genomes.org/). Large unstructured data sources, including the traditional sources such as published literature and new big data sources such as social media and electronic health records, are also now becoming part of the biomedical data domain.

The availability of these unstructured and structured data sources makes it highly desirable and feasible to query and integrate known biological information with patient-specific information. The importance of mining information from literature and combining with patient related gene expression or proteomics data has long been realized [6]. More recently, however, new unstructured data sources and new query methods are creating new biomedical insights, such as the ability to detect Flu outbreaks by mining ‘Google searches [7], [8].

Many different ways to amass, store and represent the variable data sources exist, and the intended application of the particular biomedical data store will dictate its content and structure. However, the success of any of these approaches requires a set of best practices that experimentally address both scale and performance for this type of querying. Because of the nature of the data, the appropriate choices will likely involve a combination of relational tables in conjunction with unstructured data representations. Additional application of semantics, ontologies and Natural Language Processing (NLP), to improve language interpretation and other utilities, will also be required.

As a part of a 3-month summer project, we evaluated one possible configuration for these data, using Apache Hadoop (Hadoop) (http://hadoop.apache.org/), for obtaining high performance and flexibility in querying and integrating biomedical big data. Hadoop is a widely used open source implementation of distributed computing and is being increasingly adopted in bioinformatics [9]. The project was designed to demonstrate a possible application of distributed computing technologies with two major objectives:

1. Concept/theme discovery: Searching concepts/themes in unstructured data sources such as medical literature by using standard lexicons to identify search terms through semantic expansion and perform distributed queries on the published literature to identify articles containing single terms or pairs of terms.
2. Scalability of Differentially Expressed Gene (DEG) lists to disease association: Demonstrating scalability for a huge number of structured data sets such as the gene expression or miRNA expression data sets from TCGA. Given an input set of microarray expression (or miRNA expression) data, identify a DEG list of a subset of samples against a very large set of background samples. Use the programs developed in objective (1) to create DEG to disease associations from the medical literature.

In order to accomplish the objectives, they were sub-divided into several related tasks.

The concept/theme discovery objective was sub-divided into:

- Literature counts of genes.
- Literature counts of disease terms.
- Gene – disease co-occurrence.

The scalability and DEG to disease association objective was sub-divided into:

- Identifying filtered genes from terabytes of omics data.
- Literature counts of filtered genes.
- Co-occurrence of filtered genes and disease terms.

The results of the project were graphical representations of the gene to disease and miRNA to disease networks useful to researchers attempting to understand gene-disease associations. These results form the basis for subsequent expansion to include more diverse queries in future developments.

## Results

**Figure 1:**
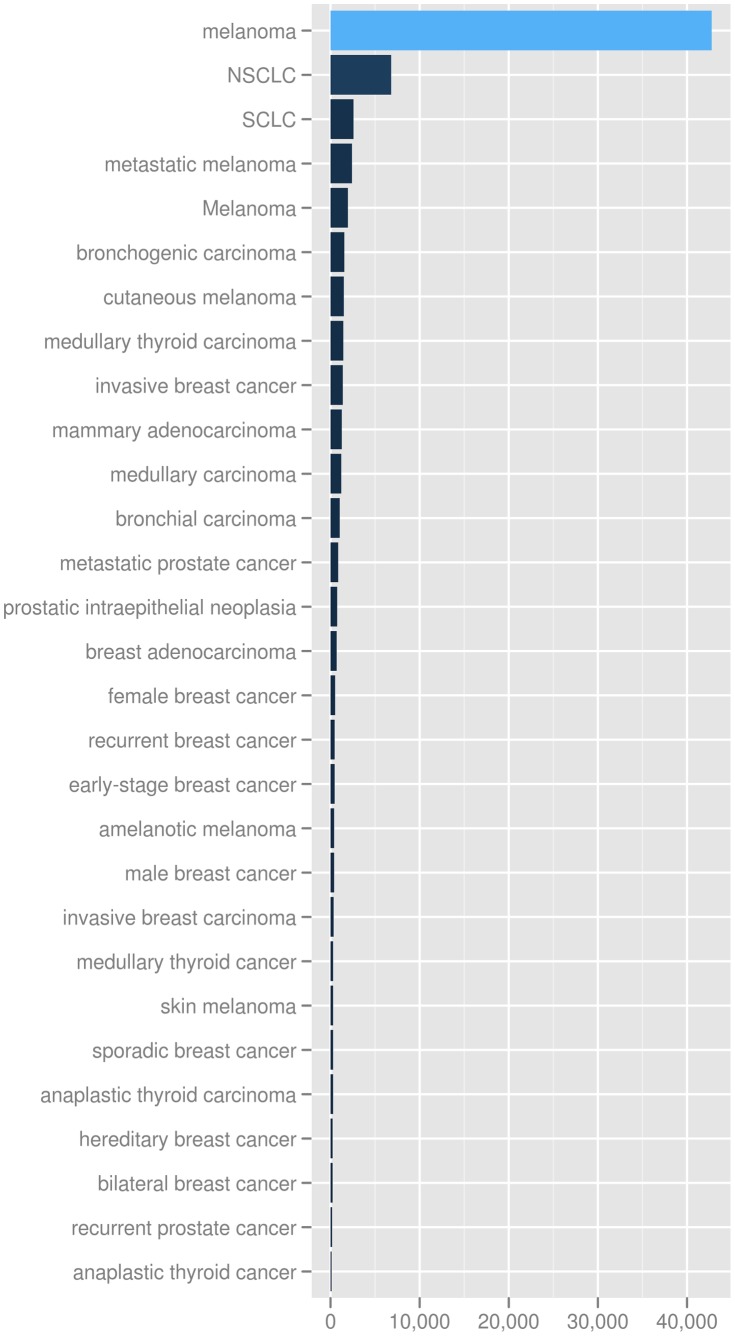
Cancer term occurrences in the literature. A bar chart representation with cancer terms on the y-axis and publication counts on the x-axis. Only the cancer terms with high literature occurrences are shown.

The test cases were intentionally engineered to align objectives with the ability to easily parallelize queries while still meeting the 12-week project timeline. All tasks were completed with high performance and tabular results were generated. All cancer terms, gene terms, miRNA terms and their corresponding PubMed ID’s are listed in tables S1, S2 and S3 respectively. The bar chart representation of the cancer term counts in literature obtained through task 1b is illustrated in Figure 1 and the gene terms are represented in Figure S1.

Lists of differentially expressed genes and miRNAs were generated after successfully completing task 2a. Performance scaled linearly when the queries were run on different sized data sets (Table 1). Tab delimited result files with counts of gene - cancer term co-occurrence and miRNA – cancer term co-occurrence generated from tasks 1c and 2c are available as tables S4 and S5. The gene – cancer term co-occurrence results were also visualized using R scripts.

**Table 1:** Load and query times using simulated gene expression data.

Task	Data Size (TB)	Datasets (millions)	Time (seconds)
HDFS Ingest	32	60	36,000
Hive Query to subset data	2	3.75	6,336
	4	7.5	7,058
	8*	15	8,304
	16	30	11,420
Hive query to extract DEGs	2	3.75	690
	4	7.5	1,347
	16	30	5,769
	32	60	10,630

* Query to get the DEG list was not run on the 8TB data due to time constraints.

Although existing literature mining methods are able to generate similar results for smaller datasets ([10]), using distributed computing and running in a batch mode decreased the time to obtain these results. As shown in Table 2, irrespective of the size of the categorical lexicon and the number of lexicons used, for each batch query, the PubMed documents are processed in their entirety in a single run giving near identical performance with different sized categorical lexicons. After the datasets were loaded, the time to query all ∼20 million abstracts with a combination of ∼11,250 genes and ∼130 disease terms i.e., a total of ∼1,450,000 term combinations, took about 1 minute with the commodity hardware-software cluster (hereto referred to as non-BDA) and 28 seconds on the Oracle Big Data Appliance (BDA). It is important to note that the result populates all cells of this 1.45M cell matrix simultaneously.

**Table 2:** Query times for batch queries on PubMed abstracts with gene, miRNA and/or cancer term lexicons.

Categorical lexicon	Number of terms	Time (seconds)
Genes	11,250	29
Cancer terms	130	28
miRNAs	530	30
Gene × Cancer terms	11,250×130	28
miRNA × Cancer terms	531×130	30

**Figure 2:**
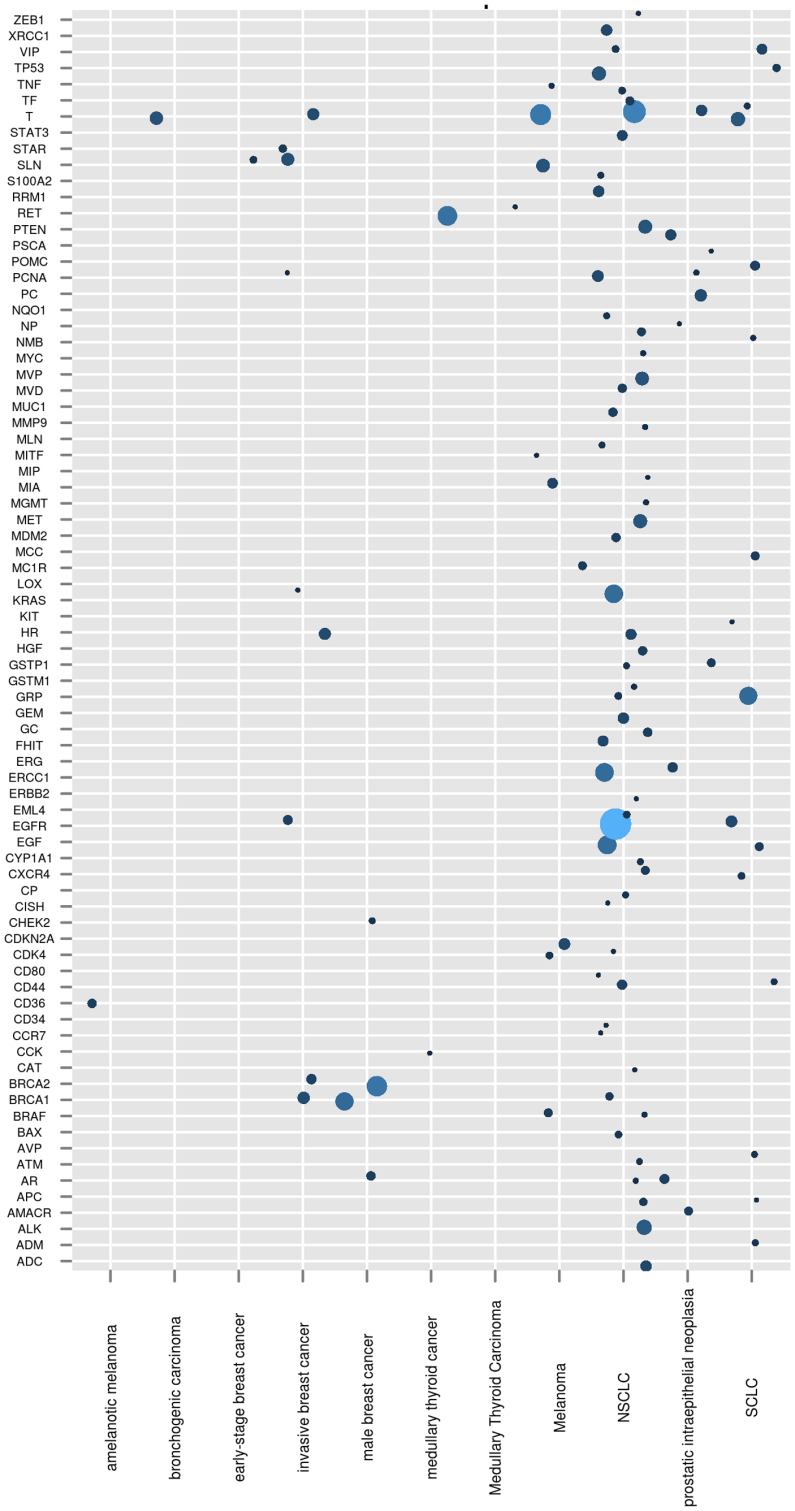
Bubble chart of Cancer-Gene associations from literature. A bubble chart representation with cancer terms on the x-axis and genes on the y-axis. The size of the bubble is directly proportional to the number of literature articles where the cancer and gene terms co-occur.

As proof that this implementation could produce biologically meaningful results, we sought to reproduce a figure from a recent issue of Genetic and Engineering News (http://www.genengnews.com/gen-articles/hotspots-of-mirna-research-activity/4162/?kwrd=miRNA). The figure in the article is a heatmap matrix with different types of cancer on one axis and specific miRNAs on the other axis. The values used for the heatmap were derived from the number of literature citations for that cancer-type - miRNA pair. R scripts were used to generate Figure 2, a similar chart to the heatmap, using genes on one axis and different cancer terms on the other axis and the number of citations represented as the size of the bubble. From the figure, it can be seen that some genes have been implicated in many cancers while other cancers seem to have fewer gene associations. Further, some genes are shared across many cancer types while others are specific to a single cancer subset. While this fact was already known, the ability to reproduce the result from a single query is very significant in being able to easily permute the query in real time.

**Figure 3:**
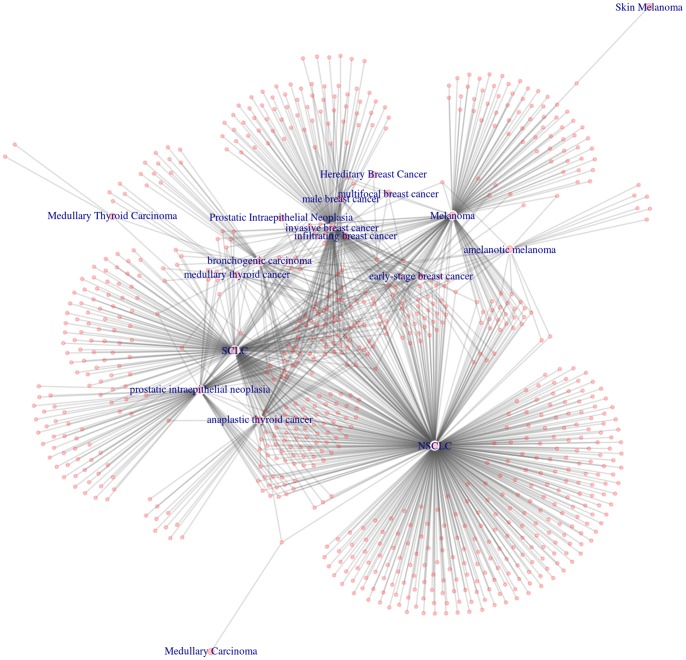
Network of Cancer-Gene associations from literature. Network of Cancer/Gene associations displaying shared genes between cancers and genes specific to certain cancer types based on literature evidence. Cancer terms are represented as labeled nodes, genes are unlabeled pink nodes and the edges represent at least one publication with a co-occurrence of the cancer term and gene.

In Figure 3, R scripts were used to generate a literature-based network of cancer term to gene linkages. This demonstrates another method to visualize the same data revealing that some genes are shared across multiple cancer types while others are unique to a particular cancer.

While the results obtained are not surprising and could be programmatically produced using PubMed (www.ncbi.nlm.nih.gov/pubmed/) or one of several literature mining tools ([11], [12]), the searches using these tools would take significantly longer as they would require selecting a subset list of genes and a subset list of disease terms and then running the query for each cell in this comparison matrix. The term combinations should also include semantic expansion of the terms to include gene aliases and disease synonyms, which would further slow and complicate the process.

The ability to dynamically produce such a graph means that clinical interpretation of mutational datasets could already be impacted by this methodology depending on the type of cancer (or other disease) and the mutational spectrum. For example, mutated genes not typically observed in a particular cancer subtype may help guide clinical treatment or even distinguish metastases from primary tumors. The results could be further evaluated for themes shared across those genes or perhaps unique to a specific subset of the genes to help guide the clinical course for that particular patient. As explained above, this scenario was demonstrated and tested through task 2c, where an actual excerpt of both mRNA and miRNA expression data was utilized and simulated data was then added to increase the data volume to test performance over a number of sample ranges extending into the millions of samples (patients). Differentially expressed gene lists could then be used to create similar networks or bubble charts of differentially expressed genes versus cancer terms.

## Discussion

In this study, methods for a scalable, dynamic data mining application that can be easily expanded to ask clinically relevant questions that retrieve literature evidence were developed. With objective 1, we derived a method to dynamically search the 20 million abstracts in PubMed using semantic expansion at impressive speeds. Both the Oracle BDA and the non-BDA cluster were able to provide the results directly through map-reduce jobs. While relatively simple language processing entities were used, more sophisticated ontologies and/or defined learning sets would presumably behave similarly and extend the value tremendously. Semantic expansion of query terms has been proven to be important in concept based searches of health conditions [13] and functional associations [14]. The ability to dynamically run millions of these queries on terabytes of unstructured documents would mean faster time to get answers that inform clinical course.

In objective 2, using prototypes of large volumes of both structured and unstructured data, it was demonstrated that queries against multiple data sources can indeed be optimized through the use of query distribution methods. The ability to integrate information from unstructured data including published literature with structured data sets including omics data, such as microarray gene expression results, is extremely powerful in the context of advancing both basic science and translational science. The importance of integrating such information and their application in understanding disease has been demonstrated previously [15], [16].

Due to the nature of the data and the analysis required, the NextGen Sequencing community has been quick to adopt distributed computing. As reviewed in the literature [9], there have been several map-reduce based software applications developed in recent years to aid in the assembly, mapping, aligning and variant analysis of the generated sequence reads [17], [18], [19]. Widely adopted bioinformatics algorithms like BLAST and GSEA have also been implemented in Hadoop [20], [21], [22] and large scale efforts have been spawned to provide consolidated knowledge bases (DOE K-Base) (http://genomicscience.energy.gov/compbio/, http://www.systemsbiologyknowledgebase.org/) and computing resources for biological researchers. This study shows that distributed computing can be leveraged to bring together structured and unstructured data sets, as the performance speeds are significant enough to influence the generation and refinement of research hypothesis in real time.

Employing these ‘Big Data’ technologies to integrate data from thousands of patients and controls might also help us understand which measurements provide the most insights into a disease mechanism. Such insights also raise the possibility that one would not need to limit data to any specific disease or other pattern and thus, similar gene expression patterns could be detected in other disease studies and these similarities would extend our understanding of commonalities in disparate disease processes.

**Figure 4:**
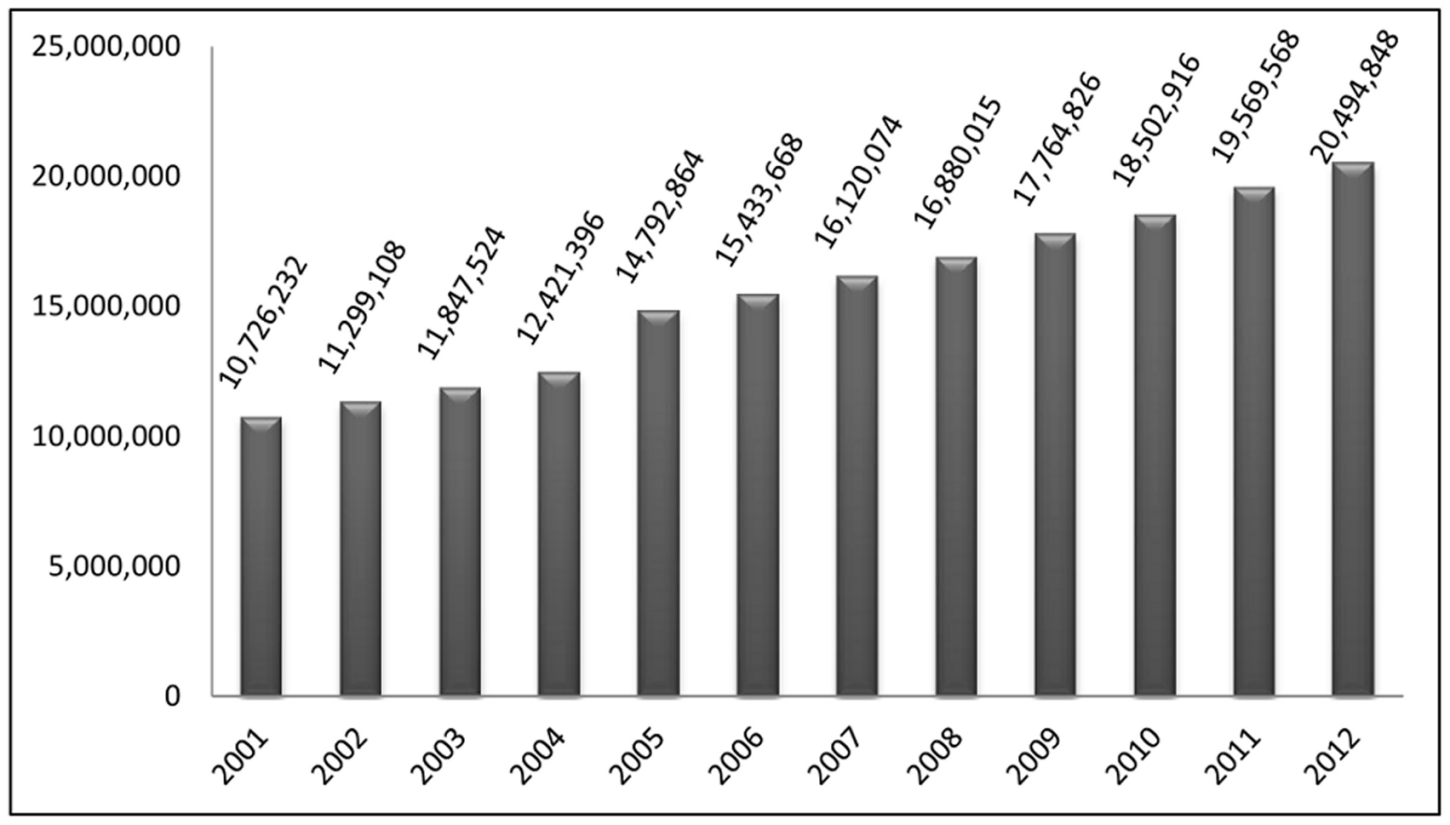
Growth of articles in MEDLINE. A bar chart displaying the number of baseline records in NLM MEDLINE’s 2001 baseline release to 2012 baseline release. (http://www.nlm.nih.gov/bsd/licensee/2012_stats/baseline_doc.html).

Although parts of the datasets used in the experiments were arguably small and the complexity of applied processing was relatively low, we believe that our test cases demonstrate the benefits of integrating distributed computing technologies as part of a broad platform on both structured and unstructured datasets. Given the initial success of the prototype, further application opportunities of the programs that were developed through this project are foreseen including:

- Expanding the prototype to integrate structured patient data sets from TCGA, including mRNA and miRNA expression, methylation and SNP data with literature and other unstructured text from sources such as ClinicalTrials.gov (http://www.clinicaltrials.gov/) and Cancer Commons (http://www.cancercommons.org/). The results obtained by integration can be stored in NoSQL data stores and be utilized for further real time comparisons with experimental data from researchers.
- Extending the program by integrating additional semantic relations and concepts derived from semantic databases like SemMedDB [23] and taking advantage of several new initiatives integrating the text mining power of Lucene with the data processing speeds obtained through Hadoop (http://incubator.apache.org/blur/, http://www.cloudera.com/content/cloudera/en/products/cdh/search.html).
- Building automated systems for annotating study-specific literature articles by using custom ontologies. Annotated literature resources like that provided by TextPresso [24] provide a huge benefit to the research community. Any performance gains associated with building such resources would be immensely helpful, especially in view of the current growth in published literature as seen in Figure 4. Study-specific vocabularies or ontologies can be used and several such lexicons combined to annotate literature for specific projects. Although these would lack the extent of functionality seen by the TextPresso application, they would still provide an immensely valuable resource for researchers, as they can be generated in a relatively short period of time and updated through automated scripts.
- Research on the applicability of integrating several omics data sets to patient stratification as observed by Yan et. al., [25]. It would be interesting to see if any of the distributed computing and NoSQL query approaches would permit querying across many patient data sets to detect disease sub-type specific gene signatures.

This pilot study demonstrated that real time integration of large structured and unstructured data sets can be achieved by leveraging massively parallel computing and querying technologies like Hadoop and Hive. The performance and results obtained with immense data sets directly translate to a greater flexibility in defining/ingesting the data sets and generating hypotheses with an exponentially faster iterative cycle time. Through the success of this project, we believe that the power of combining a distributed computing hardware platform with novel data structures and innovative query technologies has the potential to create an automated custom pipeline in which terabytes of information available from data sources like TCGA (https://tcga-data.nci.nih.gov/tcga/) and ENCODE (http://www.genome.gov/10005107) can be mined and integrated with literature evidence, using drug/disease vocabularies in any desired combination, to generate actionable biological knowledge producing improved treatment outcomes.

## Methods

Queries were performed on a commodity hardware-software cluster (non-BDA) and the Oracle Big Data Appliance (BDA) (http://www.oracle.com/us/products/database/big-data-appliance/overview/index.html). The non-BDA cluster was based upon the Sun Blade 6000 chassis. Single gigabit Ethernet interconnected 9 blades supporting 16 AMD 2224 cores, 40 Intel X5355 cores, 244 gigabytes of memory, and 1.3 terabytes of disk. The project’s BDA consisted of 18 Oracle servers interconnected by 40 Gbit Infiniband delivering 216 Intel Xeon 5675 processing cores, 2.6 terabytes of memory, and 648 terabytes of disk within a single cabinet. The disparity between the non-BDA cluster and the BDA were known from the beginning; the idea of this part of the project was not to replicate the hardware requirements of the Oracle Big Data Appliance (BDA) but to replicate the environment of the BDA so that code can be developed and queries can be run on the non-BDA cluster prior to BDA access. This was necessitated, as the BDA was accessible only during the last 2 weeks of the project.

Software applications including Java, Cloudera’s Enterprise Hadoop, Oracle R Connector for Hadoop, Oracle Loader for Hadoop, Oracle JRockit, Oracle Data Integrator, and Oracle NoSQL database were installed on the non-BDA. All these are pre-installed on and optimized for the BDA. Additional details on the non-BDA cluster are provided in document S1.

Data was acquired in a short initial phase and literature queries conforming to the objectives were performed in the later phase.

### Data Aquisition

#### Literature

Publicly available 2012 MEDLINE baseline data was licensed and downloaded from NLM (http://www.nlm.nih.gov/databases/journal.html, http://www.nlm.nih.gov/databases/license/license.html). The data consists of approximately 20 million literature abstracts formatted in XML. The size of the dataset is approximately 80 gigabytes. Along with the title and abstract of the published article, it also includes metadata about the publications such as Author, Institution, Dates, Publisher, etc. For this prototype, we were interested in three fields: PMID (The unique ID of the publication), Title and Abstract text (The abstract of the publication). In order to extract these fields, we used a simple Python script to parse each XML document and convert it to tab-delimited files containing the columns, PMID, title and abstract. The files were then ingested into the Hadoop Distributed File system (HDFS) for pre-processing. Pre-processing involved two steps: tokenizing and stop-word removal. In the first step, we did not use a standard English language tokenizer from known tools such as Lucene but instead we manually selected characters as delimiters to avoid missing Genes with symbols containing special characters such as gene Hs.6719. Next, we filtered the data to remove common English words. Since batch processing was the main goal of the prototype we elected not to use any of the KeyValue databases such as HBase or Oracle NoSQL as their main purpose is to provide low latency and near real-time response for random access while our task demands high throughput.

#### Gene lexicon

In order to create the Gene Lexicon that will be used by our program to mine the publications, we linked all Gene Symbols and Synonyms to the same Gene ID by parsing the gene_info file from EntrezGene (ftp://ftp.ncbi.nih.gov/gene/DATA/gene_info.gz). This lexicon is simply made of two fields: Gene symbol and Gene ID, where the Gene Symbol contains all human EntrezGene symbols and synonyms. For this step, we used Hive; A data warehouse system for Hadoop. Hive provides a SQL like querying language dubbed HiveQL over data residing in HDFS. Since the gene info.dat file was a tab delimited file, there was no pre-formatting and the file was loaded into HDFS and then into a Hive table. We then created the gene lexicon by extracting Gene symbols and synonyms for the Human species from the gene info table using simple queries. The created lexicon was formatted using Python and was ready to be used by our tool for mining the abstracts.

#### Disease terms

A disease lexicon containing terms related to 5 different cancers (melanoma, breast cancer, lung cancer, prostate cancer and thyroid cancer) was created. The NCI Thesaurus version 12.05d (http://evs.nci.nih.gov/ftp1/NCI_Thesaurus/archive/12.05d_ReleaseNCI) was used to create this lexicon. For this prototype, a simple lexicon was created using the terms for these 5 cancers as categories and parsing the NCI Thesaurus to look for related disease terms for each of the 5 cancers, which in some cases contained author names. As the intended goal was to test the feasibility and scalability of the approach, and due to the limitations in time, no additional processing was performed and these artifacts were treated as part of the lexicon.

#### Omics data sets

mRNA expression data and miRNA expression data from a single Glioblastoma patient, TCGA-02-0022, was downloaded from TCGA. This patient data set was chosen, since it was one of the few patients where all the data including variation results were available in the publicly available domain (https://tcga-data.nci.nih.gov/tcga/dataAccessMatrix.html ). Although all publicly available data for TCGA-02-0022 was downloaded, only the mRNA and miRNA expression data were used in this prototype.

#### Simulated data sets

To demonstrate scalability, a simulation program was created to generate the requested number of either mRNA or miRNA sample data sets. These simulated data sets were formatted based on the format of the mRNA and miRNA expression data from TCGA-02-0022. In total, 60 million simulated data files of gene expression data (32 TB) and 900 million simulated data files of miRNA expression data (18 TB) were generated. The simulated data was created such that the approximate distribution of expression values and values range were reproduced for each sample from randomly generated values.

### Queries

To achieve the objectives we developed a program for tagging text documents using a categorical lexicon. The lexicon consists of a list of tokens and their corresponding categories. A category can consist of several tokens. Also a token can be associated with more than one category. The use of these categorical lexicons is not limited to the scope of this project but can be used in sentiment analysis, feature extraction and other areas. We used categorical lexicons to associate the variations of gene symbols with their corresponding gene IDs. For example, gene symbols ACTG1P1, ACT1GP1, ACTGP1 and HY-psi-gamma-AC6 all correspond to one gene. The program parses each document by tokenizing the text into tokens then uses the categorical lexicons to look for the appearances of terms within the document and tagging the document with the corresponding categories. The program is also capable of scanning multiple tokens at once (Window of tokens). This capability was needed since the disease lexicon contained multiple-word diseases such as “Invasive Breast Carcinoma”. We used Java for developing the code and implemented the MapReduce framework as the distributed processing model for scalability. Each mapper processes a single document and outputs the document ID as key and the associated categories as value.
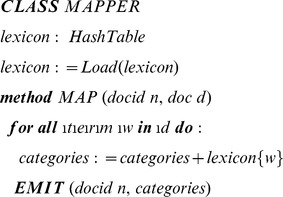



### Objective 1: Concept/Theme Discovery

#### a) Literature count for genes

We processed the abstracts with a map-reduce job using the gene categorical lexicon to tag each publication with the list of gene IDs it contained. The results were also grouped to get the number of abstracts containing each of the human genes annotated in EntrezGene.

#### b) Literature count for disease terms

The cancer based disease categorical lexicon was used for getting the literature associated with all 5 cancers. The results were the counts of the number of abstracts containing each cancer term.

#### c) Gene – Disease co-occurrence query

In order to look for co-occurrences, we extended our program to take two categorical lexicons and output the co-occurrences of categories as pairs, as well as the number of abstracts containing them.
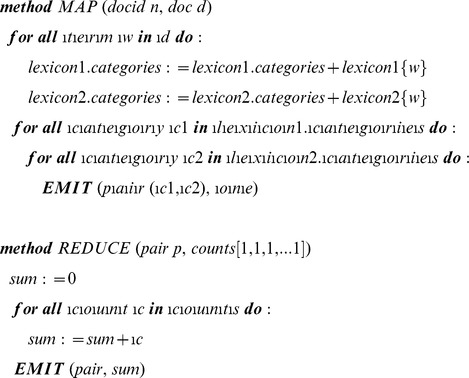



The program was used to query for the co-occurrence of every possible gene-cancer term combination derived from the gene and cancer lexicons. R scripts were used to visualize the co-occurrence results as bubble charts and network views.

### Objective 2: Scalability and DEG to Disease Association

#### a) Identifying Differentially Expressed Genes (DEGs)

Simulated gene expression and miRNA expression datasets, generated based on sample TCGA data sets from a single patient, were loaded into HDFS. Due to the differences in the two clusters, the data set sizes varied between the non-BDA and the BDA, and scalability and performance testing for larger data sets was limited to the BDA (Table 1). Only a limited simulated data sets, roughly equivalent to 100 thousand mRNA expression and 1 million miRNA expression data sets were used in the non-BDA while it was scaled to relatively large data sizes of 60 million mRNA expression and 900 million miRNA expression data sets for the BDA. In total, 32TB of gene expression and 18TB of miRNA expression data, were ingested into HDFS in the BDA. Simple ‘create table’ Hive queries were used to generate subsets of the data. Hive queries with expression level filter of log2x, to get differentially expressed genes/miRNAs with greater than 2 fold expression, were run on these subsets.

#### b) Literature counts of DEGs

Tables with the differentially expressed genes/miRNAs and the counts of the number of abstracts in which these terms occur were generated using the map-reduce jobs created through objective 1a.

#### c) Co-occurrence of filtered genes and disease terms

The extended version of the program described in objective 1c, in combination with R was used to create co-occurrence graphs for differentially expressed genes and disease terms.

**Figure 5:**
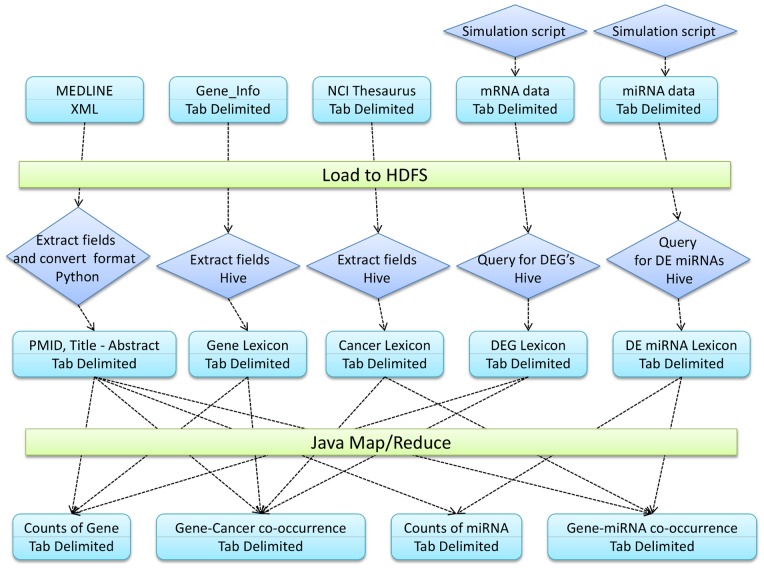
Architecture for integrating structured and unstructured data in Hadoop. Architectural diagram detailing the steps in creating the categorical lexicons and using them to get the PMID counts from literature. DEG stands for Differentially Expressed Gene while DE miRNA stands for Differentially Expressed miRNA.

The different tasks and steps involved in achieving the objectives are shown in Figure 5.

## Supporting Information

Figure S1 **Gene term occurrences in the literature.** A bar chart representation with genes on the y-axis and publication counts on the x-axis. Only the genes with high literature occurrences are shown.
(TIFF)

Table S1 **Cancer term occurrences in the literature.** A three column tabular representation with cancer terms, number of publications and the PubMed ID’s of articles containing the cancer term.
(TXT)

Table S2 **Gene occurrences in the literature.** A three column tabular representation with genes, number of publications and the PubMed ID’s of articles containing the gene term.
(TXT)

Table S3 **miRNA occurrences in the literature.** A three column tabular representation with miRNAs, number of publications and the PubMed ID’s of articles containing the miRNA term.
(TXT)

Table S4 **Gene-Cancer Co-occurrences in the literature.** A four column tabular representation with genes, cancer terms, number of publications and the PubMed ID’s of articles containing both the gene and cancer terms.
(TXT)

Table S5 **miRNA-Cancer Co-occurrences in the literature.** A four column tabular representation with miRNAs, cancer terms, number of publications and the PubMed ID’s of articles containing both the miRNA and cancer terms.
(TXT)

Document S1 **non-BDA cluster.** A document detailing the creation of the non-BDA cluster and some performance metrics.
(DOCX)
