# Supplementary figures and images for: Knowledge and Theme Discovery across Very Large Biological Data Sets Using Distributed Queries: A Prototype Combining Unstructured and Structured Data
Source: PLoS One. 2013 Dec 2;8(12):e80503. doi: 10.1371/journal.pone.0080503 (PMC3846626; doi:10.1371/journal.pone.0080503)

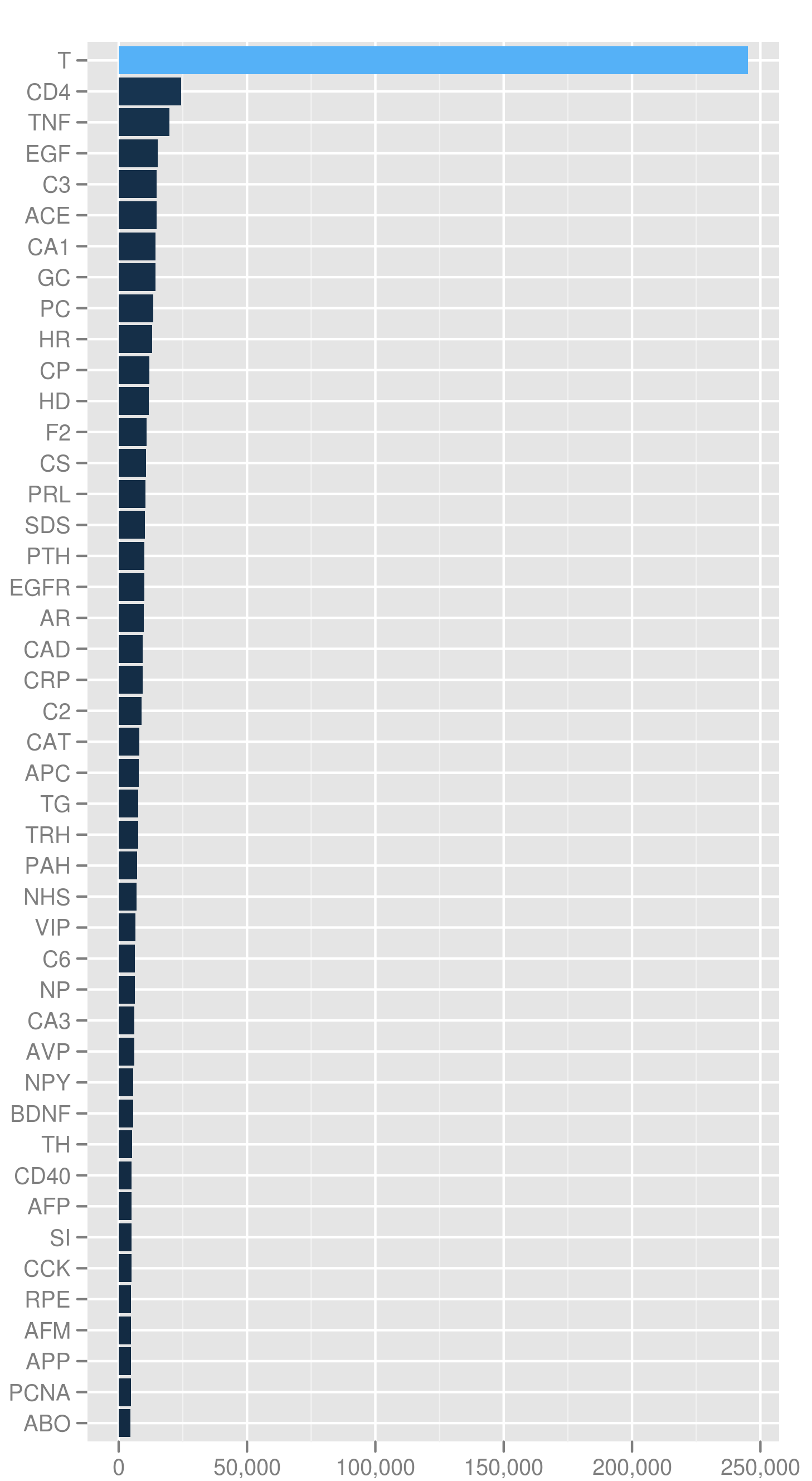

Supplement: Figure S1 — Gene term occurrences in the literature. A bar chart representation with genes on the y-axis and publication counts on the x-axis. Only the genes with high literature occurrences are shown. (TIFF) [file pone.0080503.s001.tiff]
