# Supplementary material for: Knowledge and Theme Discovery across Very Large Biological Data Sets Using Distributed Queries: A Prototype Combining Unstructured and Structured Data
Source: PLoS One. 2013 Dec 2;8(12):e80503. doi: 10.1371/journal.pone.0080503 (PMC3846626; doi:10.1371/journal.pone.0080503)
Supplement: Document S1 — non-BDA cluster. A document detailing the creation of the non-BDA cluster and some performance metrics. (DOCX) [file pone.0080503.s007.docx]

Non-BDA cluster

The non-BDA cluster was used for replicating the environment on BDA, so that queries can be run prior to BDA access. To form the cluster, 5 Sun Blade 6250s with (2) Intel X5355 CPUs and 32GB RAM, and 4 Sun Blade 6220s with (2) AMD 2224 CPUs and 16GB RAM with a total of 56 CPU cores and 224GB RAM were used. Each node except for one, with 400GB internal disk utilized for the Hadoop data storage cluster, had a 146GB SAS data disk.

The blades were imaged with Oracle Enterprise Linux 5.6 and connected to each other by a 1Gbps copper network backbone. The Cloudera Manager was installed on one of the AMD blades, and the client was installed on the other 8 blades. Several software including including Java, Cloudera’s Enterprise Hadoop, Oracle R Connector for Hadoop and Oracle Loader for Hadoop were installed. The installation of the cluster up through the Apache software took approximately (4) days, including the time it took to image nodes, install the Cloudera suite and software, and complete the configuration of the software so that processing could be performed upon the Hadoop/MapReduce cluster. An additional 2 days were required for the installation of the Oracle software, reading documentation, and troubleshooting errors and warning messages. The hardware used for the Non-BDA experimental setup was already in a rack and cabled, otherwise it would have likely added several hours to the setup time.

The TeraSort Hadoop application was run on the non-BDA and BDA to setup some base benchmarks but otherwise no detailed performance comparisons were performed. The application was run against a dataset of 100GB which was sized based on the storage we had in the non-BDA. In our testing, we found that with 156 map tasks for data generation via TeraGen and 39 reduce tasks for the TeraSort process, the data was sorted at a speed of 55MBps in the non-BDA and 1.11GBps in the BDA. The lower than anticipated speed on the non-BDA is most likely due to having only a single data disk in each of the processing nodes. The single-disk nodes used in our cluster were not optimized for data writing, and this is what we think decreased the speed of the overall processing.

Additional benchmarks were performed on the BDA varying the number of reduce threads and the size of the data sets. Most variability was observed with changes in the number of reduce threads, where TeraSort tests using 26 reduce threads on a 1TB dataset ran at 1.06GBps while the job with 150 reduce threads ran at 2.34GBps.

In order to view the overall status of the non-BDA cluster, we utilized *Ganglia*. This software created real-time graphs and charts of cluster resources, including CPU and memory usage.


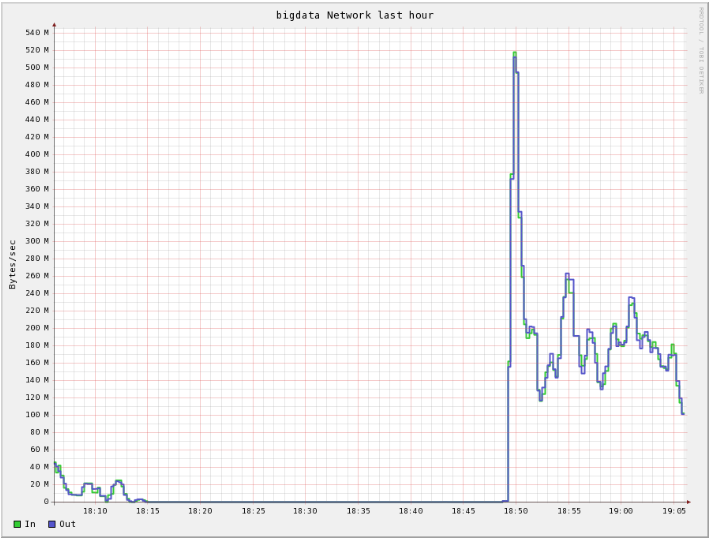


Fig 1: non-BDA network usage during 100GB terasort

Given the dataset size we worked with, on the non-BDA, we were not in dire need of anything more than a 1Gbps network backbone. In Fig 1, we see that the maximum network usage for the 100GB dataset was only around 500Mbps and we anticipate that the usage would be much higher for larger datasets.


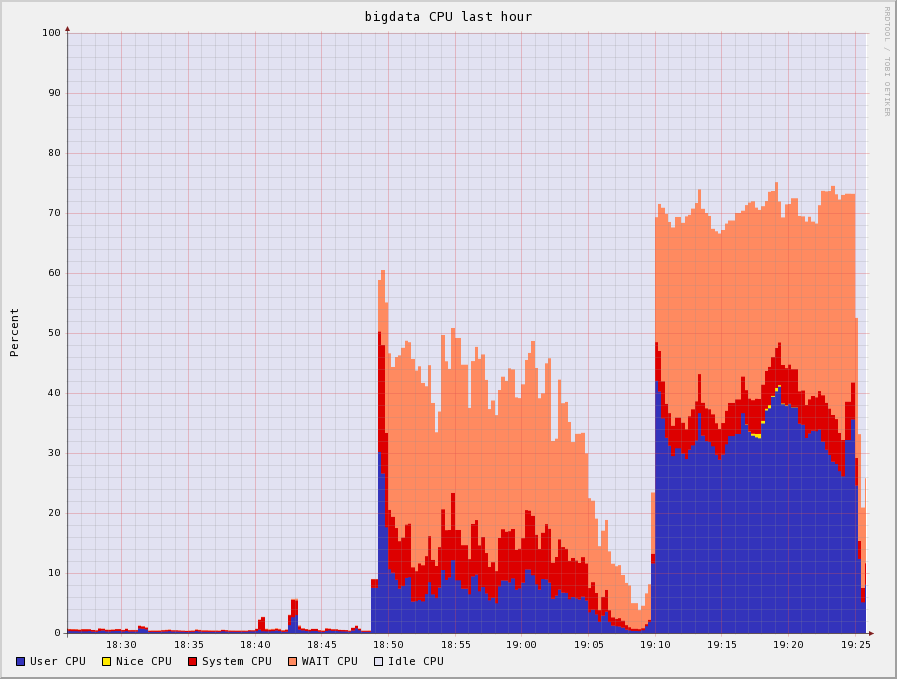


Fig. 2: non-BDA CPU Usage During 100GB TeraSort

The Ganglia performance monitor showed that during the TeraSort tests, 30-40% of the CPU time was spent on I/O wait - in this case, reading and writing the data to disk (Fig.2). It also appears that due to the shuffling process of MapReduce, which merges processing outputs from memory to disk, performance can degrade when disks are close to capacity. When the disk on a processing node is full, the task attempting to write data to the disk must be re-run on another disk with more space so that the output can be recorded. This is a time consuming process that reduces performance.

Memory was also a factor in the setup of our non-BDA, but it was not a limiting factor for most of our tests. The more resource-intensive tests used much of the RAM in the system but none were using it to the maximum limit (Fig. 3).


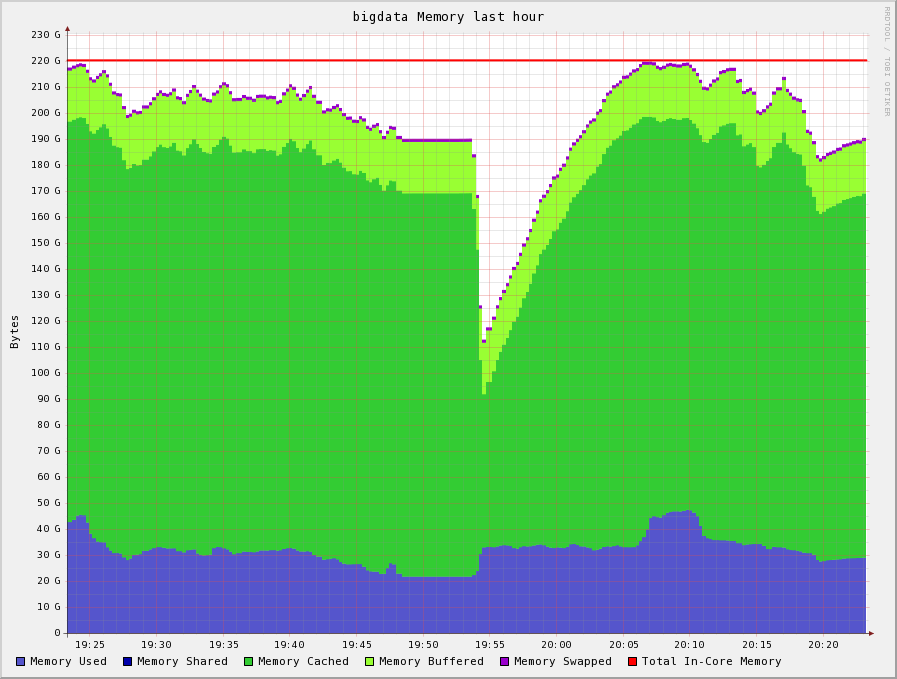


Fig 3: NBDA Memory Usage During TeraSort
